# Acceptability of telemedicine for follow up after contraceptive implant initiation at an obstetrics and gynecologic training center

**DOI:** 10.1186/s12913-023-09816-7

**Published:** 2023-07-31

**Authors:** Jarika Vatrasresth, Peerapong Prapaisilp, Monchada Sukrong, Natchanika Sinthuchai, Parichart Karroon, Duangporn Maitreechit, Sirarat Ittipuripat, Arissara Kuptarak, Sarochinee Sathitloetsakun, Somsook Santibenchakul, Unnop Jaisamrarn

**Affiliations:** 1 Department of Obstetrics and Gynecology, Faculty of Medicine, Chulalongkorn University, Bangkok, Thailand; 2 Faculty of Medicine, Chulalongkorn University, Bangkok, Thailand; 3 Department of Epidemiology, Harvard T.H. Chan School of Public Health, Harvard University, Boston, MA, USA; 4 Outpatient unit Gynecology and Family planning, King Chulalongkorn Memorial Hospital, Bangkok, Thailand; 5 Department of Nursing, KCMH lactation clinic, King Chulalongkorn Memorial Hospital, Bangkok, Thailand; 6 Department of Obstetrics and Gynecology, University of Phayao Hospital, Phayao, Thailand; 7 Department of Obstetrics and Gynecology, King Chulalongkorn Memorial Hospital, Bangkok, Thailand

**Keywords:** Telemedicine, Telehealth, Contraceptive implant, Long-acting reversible contraceptives, COVID-19

## Abstract

**Background:**

During the COVID-19 pandemic, telemedicine has become a popular adjunct to in-person visits, including for family planning services. This study determined the proportion of clients participated in telemedicine services and the association between sociodemographic factors and telemedicine participation during the COVID-19 pandemic. The adverse effects within the first seven days post-insertion were also reported.

**Methods:**

This retrospective cohort study considered data from all women initiating contraceptive implantation between June 2020 and August 2021 at King Chulalongkorn Memorial Hospital. Clients were offered the following two options for follow-up visits: in-person or communication via an online LINE® Official Account (LINE® OA), a free chat application widely used among the Thais. Logistic regression analyses were used to assess the association between socio-demographic factors and telehealth usage.

**Results:**

In total, 574 of 947 (60.6%) clients participated in telemedicine follow-up services during the period considered. A significant association between telemedicine usage and the following were observed: the peak of second wave COVID-19 outbreak in Thailand, using the period preceding the second wave as a reference [adjusted odds ratio (aOR) = 1.47 (95% confidence interval [CI]: 1.12–1.96)]; participants receiving governmental benefits for contraceptive implant payment (aOR: 3.23, 95% CI: 1.86–5.60), and timing of contraceptive implant(s) initiation, using interval insertion as a reference for which aORs of postpartum and immediate postpartum insertions were 0.62 (95% CI: 0.43–0.90) and 0.35 (95% CI: 0.24–0.52), respectively. Significant ecchymosis at the insertion site was observed in 13.1% of participants.

**Conclusion:**

This study emphasizes the significance of telemedicine during the COVID-19 epidemic, particularly in facilitating contraceptive implant initiation. Our data show a significant increase in the uptake and utilization of telemedicine during the pandemic’s peak. The data also shows that during the period of Thailand’s second COVID-19 epidemic, government benefits for contraceptive implant payment, and the timing of contraceptive implant initiation, are significantly associated with telemedicine use. This finding supports the continued use of telemedicine in healthcare, particularly for services like family planning, where remote follow-ups can provide safe, efficient, and timely care.

## Background

Subdermal contraceptive implants are long-acting reversible contraceptives (LARC) that provide excellent contraceptive efficacy [1, 2]. In the past, all contraceptive devices were self-paid, regardless of the reimbursement method. Since 2014, the Thai government has launched a policy that aims to reduce unintended pregnancy. Adolescents, women who undergo safe abortion services, and those living in Bangkok may receive a contraceptive implant or a copper intrauterine device at no or low cost. Individuals covered by certain reimbursement methods, such as universal coverage or government officers, are exempted from additional service fees, which amount to approximately $3. Contraceptive implant initiation has increased dramatically, especially among adolescents and women who have undergone abortions [3–5]. However, side effects contribute to early implant discontinuation [6–8]. Providing adequate counseling before contraceptive initiation and continuing support from healthcare providers may mitigate the discontinuation rate among some groups of individuals such as adolescents and young women which make up the majority of our clients.

Proper training of providers is important for improving contraceptive implant services [9–11]. Though contraceptive implant insertion and removal are elective and minor procedures; meticulous surgical techniques are important for improving client safety and satisfaction. Feedback modalities are essential for training providers [12] and evaluating post-procedure outcomes a key feedback system used for improving the skill level of trainees. The Family Planning and Reproductive Health Clinic of King Chulalongkorn Memorial Hospital (KCMH) is the primary outpatient training site of Obstetrics and Gynecology residents completing rotations. At the clinic, we recommend one-week and three-month post-insertion follow-up visits for all women receiving contraceptive implants.

During the global health crisis of the COVID-19 pandemic, the healthcare system faced significant challenges. Social distancing policies aimed at reducing the transmission of virus, have necessitated the suspension of both public and private activities, consequently impacting medical training and services. The burden of high COVID-19 caseloads further strained the healthcare system, pushing it to its limits and exacerbating the challenges faced in delivery timely and adequate healthcare services. As a result, non-urgent care and elective procedures, including family planning and contraception services, were postponed. Since June 2020, after the initial outbreak of COVID-19 in Thailand, the Family Planning and Reproductive Health Clinic has offered the following two client options for post-insertion follow-up visits: in-person or telemedicine. For telemedicine follow-up services, we selected the LINE® Official Account (LINE® OA) platform, a free chat application that is popular among Thai people. Our study aimed to determine the decision to participate in telemedicine services and the association between sociodemographic factors and telemedicine usage during the COVID-19 pandemic. In addition, we reported adverse effects of contraceptive implant initiation during a 7-day post-insertion period.

## Methods

This was a retrospective cohort study that used data collected from all women initiating contraceptive implantation during the COVID-19 pandemic (June 2020–August 2021) at KCMH, Thailand, a tertiary care hospital and obstetrics-gynecology residency training center from which approximately 800 to 1000 cases initiate contraceptive implantation each year. All women who received contraceptive implant services at KCMH during this period were included. Our service policy during the pandemic was adapted to that of the Thai government, including decreasing numbers of outpatient contraceptive implant initiation cases per day or postponing follow-up visits.

Before the COVID-19 pandemic, it was clinic policy to offer all clients a seven-day post contraceptive initiation visit. However, to comply with the social distancing policy of the Thai government, since June 2020, a telemedicine-based option for follow-up services has been provided at no additional cost. As a result, women have voluntarily participated in the service. Participants deciding to return for an in-person visit were scheduled to return to the clinic. Those deciding to be followed-up via a telemedicine-based visit were offered a QR code to access the LINE® OA system. After logging into the LINE® OA platform, an automated message regarding registration for post-insertion services was sent to all participants. All participants were asked to send photos of the implant site at 7-days post-insertion. All participants were offered post-insertion counseling services through the LINE® OA platform in which participants were able to exchange text, audio, and video messages with the study team. Most concerns of participants were addressed via text message by the study team within 24–48 h. In the present study, participants were followed-up for a 7-day period post-insertion.

The medical consultation team, comprising two doctors and two nurses, assessed clients’ histories and reviewed implantation site photos. We provided personalized management recommendations, including self-care options and the option to return for in-person visits if needed. Self-management techniques such as dressing changes, cold compression, and pain relief medication were advised as appropriate. For any complicated questions or adverse events, nurses were required to consult a doctor before responding to the client. Baseline characteristics of study participants were collected from electronic medical records. Adverse events occurring during 7-day periods following insertion were reported from the chat records. Participant characteristics considered included age, type of contraceptive implant(s), timing of contraceptive implant(s) initiation, type of payment, number of parity, and underlying disease. In addition, characteristics of women who did not participate in telemedicine services were collected from electronic medical records detailing contraceptive implant initiation visits.

In the first seven-day assessment of telemedicine services, data regarding adhesive-related complications were collected and divided into three categories, as follows: bleb or rash, bleeding and irritation, and insertion site complications such as ecchymosis at the implantation site, with significant ecchymosis (defined as an area of discoloration larger than 2 × 2 cm). The presence of pain, redness, swelling, and numbness at the surgical site or ipsilateral arm were also recorded. Implant initiation times were divided into two periods, June 2020–November 2020 and December 2020–August 2021, which corresponded to the timing of before and during the second wave of the COVID-19 pandemic in Thailand, respectively [13]. Considering that the pandemic itself may have influenced the adoption of telemedicine, we selected the peak of COVID-19 cases as a distinct cut-off point in our timeline. This was intended to allow for a more accurate analysis of how the pandemic severity may have influenced telemedicine usage.

Data were collected using Research Electronic Data Capture (REDCap) [14, 15] tools and placed in a spreadsheet for analysis using IBM SPSS Statistics for Windows, version 22.0 (IBM Corp., Armonk, NY, USA). Descriptive data are shown as a frequency and percentage for categorical variables, and mean and standard deviation (SD) for numerical variables. Unpaired t-tests and Chi-square tests were used to assess differences between groups. Univariable and multivariable logistic regression were used to assess the association between socio-demographics and telehealth usage. We selected variables as potential confounders based on criteria from existing literature, which included either clinical relevance or statistical significance demonstrated within a univariable model. A backward logistic regression model was used to control for the potential confounders. Statistical significance was set at p < 0.05. This study was approved by the Institutional Review Board of the Faculty of Medicine, Chulalongkorn University (IRB601/64).

## Results

Between June 2020 and August 2021, 947 women initiated contraceptive implantation at the KCMH. Among them, 574 (60.6%) opted to participate in telemedicine follow-up services. The mean (SD) ages of patients in telemedicine (27.3 ± 7.4 years) and in-person (27.3 ± 7.6 years) groups did not statistically differ (p > 0.05). Almost half of participants from both groups (43.5%) were aged 25–35 years. Approximately one-third of participants have never had children. The interval insertion accounted for the majority of initiations, with 39.0% (369/947) of participants, followed by postpartum initiations (21.1%). Approximately 19% (177/947) of study participants were continuous users. Some participants [7.7%] paid for their contraceptive implants out of their pockets. Most (85.6%) had no underlying medical conditions. Before the peak of the COVID-19 pandemic (June 2020–November 2020), 236 of 431 (54.7%) participants used telemedicine services. During the peak of the COVID-19 pandemic (December 2020–August 2021), 338 of 516 (65.5%) participants used telemedicine services (p < 0.05, Table 1).

**Table 1:** Basic characteristics (N = 947)

Variables	Telemedicine-based n = 574 n (%)	In-person n = 373 n (%)	Total N = 947 n (%)
Age (mean [SD])	27.3 (7.4)	27.3 (7.6)	27.3 (7.5)
Age			
▪ ≤ 19	91 (15.9)	54 (14.5)	145 (15.3)
▪ > 19–24	151 (26.3)	88 (23.6)	239 (25.2)
▪ > 24–35	238 (41.5)	174 (46.6)	412 (43.5)
▪ > 35–45	82 (14.3)	52 (13.9)	134 (14.1)
▪ > 45	12 (2.1)	5 (1.3)	17 (1.8)
Duration of initiation			
▪ Before the peak of COVID-19 (June 2020 – November 2020)	236 (41.1)	195 (52.3)	431 (45.5)
▪ During the peak of COVID-19 (December 2020 – August 2021)	338 (58.8)	178 (47.7)	516 (54.5)
Type of contraceptive implant(s)			
▪ Etonogestrel (Nexplanon®)	329 (57.3)	229 (61.4)	558 (58.9)
▪ Levonorgestrel (Jadelle®)	245 (42.7)	144 (38.6)	389 (41.1)
Type of payment			
▪ Government benefits	543 (95.6)	321 (87.2)	864 (92.3)
▪ Self-payment	25 (4.4)	47 (12.8)	74 (7.7)
Timing of initiation*			
▪ Postpartum	121 (21.1)	80 (21.4)	201 (21.1)
▪ Immediate postpartum	96 (16.7)	104 (27.9)	200 (21.1)
▪ Interval	249 (43.3)	120 (32.2)	369 (39.0)
▪ Interval changing new contraceptive implant(s)	108 (18.8)	69 (18.5)	177 (18.7)
Parity			
▪ 0	215 (37.5)	114(30.6)	329 (34.8)
▪ 1	192 (33.5)	127 (34.0)	319 (33.7)
▪ 2	113(19.7)	80 (21.4)	193 (20.4)
▪ ≥ 3	53 (9.2)	52 (13.9)	105(11.1)
Number of living children			
▪ 0	218 (38.0)	116 (31.1)	334 (35.3)
▪ 1	192 (33.4)	128 (34.3)	320 (33.8)
▪ 2	114 (19.9)	81 (21.7)	195 (20.6)
▪ ≥ 3	50 (8.7)	48 (2.9)	98 (10.3)
Reimbursement			
▪ Universal health Coverage	134 (24.5)	108 (30.9)	242 (27.0)
▪ Social Security Scheme	176 (32.2)	110 (31.4)	286 (31.9)
▪ Health insurance	3 (0.5)	0 (0)	3 (0.3)
▪ Cash payment	168 (30.8)	91 (26.0)	259 (28.9)
▪ KCMH** officer	40 (7.3)	24 (6.9)	64 (7.1)
▪ Government/ State enterprise officer	25 (4.6)	17 (4.9)	42 (4.7)
Underlying disease			
▪ No	506 (88.2)	313 (83.9)	819 (86.5)
▪ Yes (at least one condition)***	68 (11.8)	60 (16.1)	129 (13.5)

*Postpartum: This refers to the delayed insertion of the contraceptive implant during the first postpartum visit, typically around six weeks after childbirth. Immediate postpartum: This involves the insertion of the contraceptive implant shortly after childbirth. Interval insertion: This refers to the insertion of the contraceptive implant at any time during the menstrual cycle, independent of pregnancy status. Interval change of contraceptive implant(s): This involves the replacement of an existing contraceptive implant with a new one

**King Chulalongkorn Memorial Hospital

***Telemedicine-based: Hematologic disease (n = 14), Thyroid disease (n = 6), Cardiovascular disease (n = 11), Neurologic disease (n = 5), Rheumatologic disease (n = 3), Psychiatric disease (n = 17), In-person: Hematologic disease (n = 6), Thyroid disease (n = 5), Cardiovascular disease (n = 4), Neurologic disease (n = 5), Rheumatologic disease (n = 2), Psychiatric disease (n = 18)

The association between participant characteristics and telemedicine usage was analyzed. During the peak of the COVID-19 pandemic in Thailand, the payment method for the contraceptive implant, timing of contraceptive implant initiation, and number of living children were significantly associated with the use of telemedicine services via univariable logistic regression analyses, as shown in Table 2. Univariable logistic regression analysis revealed that the peak of the COVID-19 pandemic in Thailand was associated with increased usage of telemedicine services, with results of the multivariable logistic regression analysis slightly attenuated the effect size. Compared to the period preceding the second wave of COVID-19, the adjusted odds ratio (aOR) and 95% confidence interval (CI) for telemedicine participation for the peak of second wave were 1.47 and 1.12–1.96, respectively. Compared to paying for the contraceptive implant out of pocket, the aOR value for the association between telemedicine participation and eligibility for government benefits was increased (aOR: 3.23, 95% CI: 1.86–5.60). The timing of the initiation of contraceptive implants was also significantly associated with the use of online services via multivariable logistic regression analysis. Compared to interval insertion, contraceptive implant insertion during the immediate postpartum and postpartum period and interval changing the new one had lower aOR values for telemedicine participation of 0.35 (95% CI: 0.54–0.52), 0.62 (95% CI: 0.43–0.90), and 0.68 (0.46–1.01), respectively. Although the presence of underlying disease showed a trend towards lower odds ratio for telehealth usage, the association was not statistically significant. No association between age group, type of contraceptive implant initiation, or type of reimbursement was demonstrated (Table 2).

**Table 2:** The association between participants’ demographics and telehealth usage participation. (N = 947)

Variables	OR (95% CI)	p-value	adjusted OR (95% CI)	p-value
Age				
▪ < 24	1.03 (0.69–1.53)	0.41		
▪ > 24–35	0.85 (0.58–1.24)			
▪ > 35	reference			
Duration of initiation				
▪ Before the peak of COVID-19 (June 2020 – November 2020)	reference		reference	
▪ During the peak of COVID-19 (December 2020 – August 2021)	1.56 (1.20–2.04)	< 0.01	1.47 (1.12–1.96)	< 0.01
Type of contraceptive implant(s)				
▪ Etonogestrel (Nexplanon®)	0.84 (0.65–1.10)	0.21		
▪ Levonorgestrel (Jadelle®)	reference			
Type of payment				
▪ Government benefits	3.18 (1.92–5.27)	< 0.01	3.23 (1.86–5.60)	< 0.01
▪ Self-payment	reference		reference	
Timing of initiation				
▪ Postpartum	0.73 (0.51–1.04)	< 0.01	0.62 (0.43–0.90)	< 0.01
▪ Immediate postpartum	0.44 (0.31–0.63)		0.35 (0.24–0.52)	
▪ Interval changing new contraceptive implant(s)	0.75 (0.52–1.09)		0.68 (0.46–1.01)	
▪ Interval	reference		reference	
Number of living children				
▪ 0	reference			
▪ ≥ 1	0.74 (0.56–0.97)	0.03		
Reimbursement				
▪ Universal health Coverage	reference			
▪ Social Security Scheme	1.29 (0.91–1.83)	0.18		
▪ Cash payment	1.49 (1.04–2.13)			
▪ Other*	1.34 (0.84–2.12)			
Underlying disease				
▪ No	reference			
▪ Yes	0.70 (0.48–1.02)	0.06		

*Other: Health insurance, King Chulalongkorn Memorial Hospital officer, and Government/ State enterprise officer

**Fig. 1:**
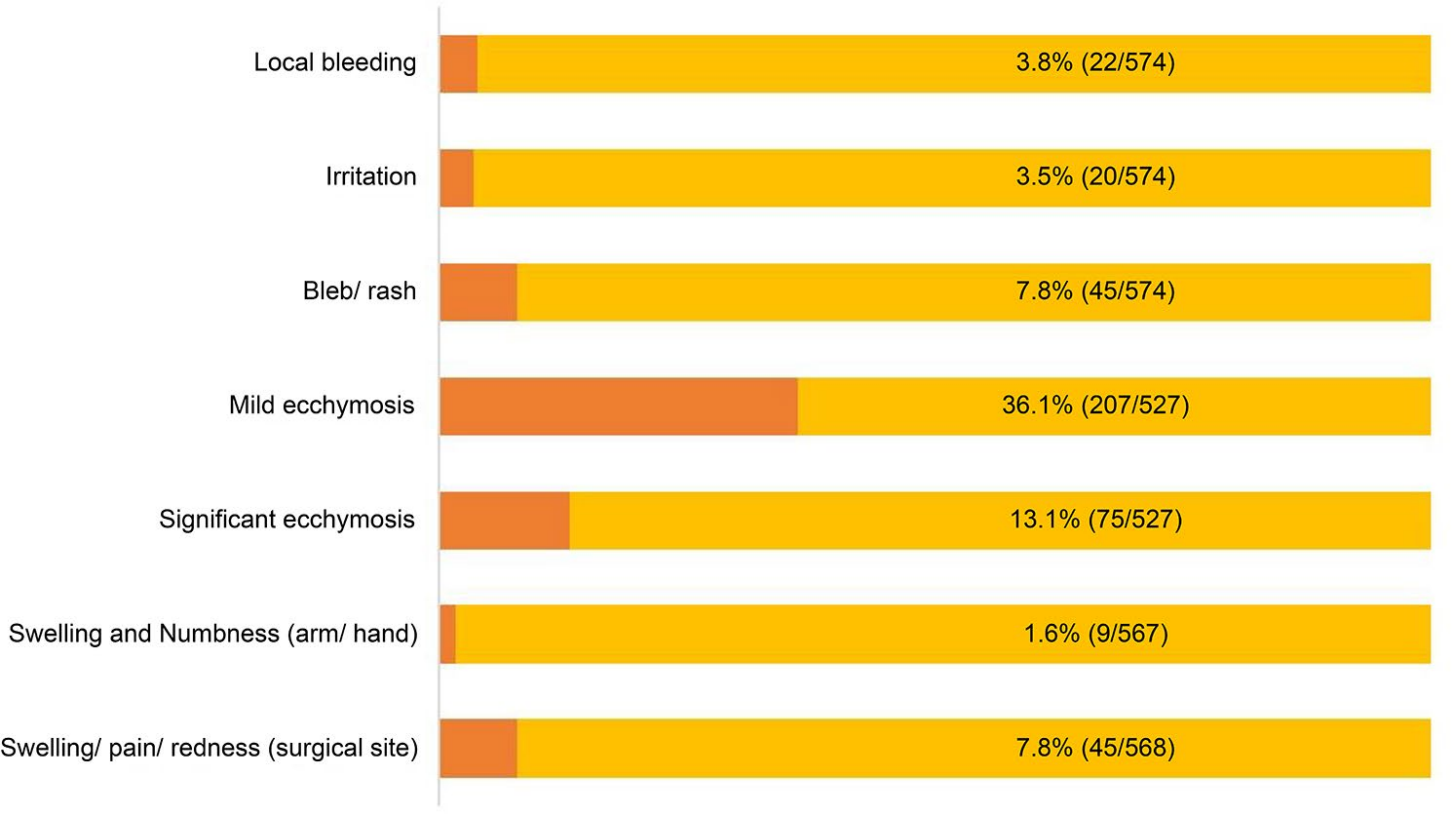
Adverse effects assessment within the first seven days after contraceptive implant(s) initiation among participants who decided to undergo telehealth follow-up services via Line Official application. [n = 574])

During the first seventh day after initiation, participants using the online platform for follow-up were asked to send pictures of the implant insertion site to facilitate an evaluation of the local site. Blebs or rash, bleeding, and irritation were found in 7.8%, 3.8%, and 3.5% of participants, respectively. Significant ecchymosis at the initiation site was observed in 13.1% of participants. Some participants (7.8%) experienced local reactions including pain, redness, or swelling at the surgical site (Fig. 1).

## Discussion

Approximately 60% of clients participated in telemedicine follow-up services during the COVID-19 period. Telemedicine usage was found to be significantly associated with the peak of the second wave of COVID-19 in Thailand. Participants who received governmental benefits for contraceptive implant payment were more likely to use telemedicine. The timing of contraceptive implant initiation was also associated with telemedicine usage, with postpartum and immediate postpartum insertions having lower odds ratios compared to interval insertions. A few participants experienced minor adverse events such as bleeding at the surgical site, skin bleb, or rash. Approximately 15% of participants had significant ecchymosis at the surgical site.

Contraceptive implants are highly effective and safe for preventing unintended pregnancies, especially among adolescents and young adults. Although the Centers of Disease Control and Prevention (CDC) recommend against a requirement for routine follow-up after contraceptive initiation due to a lack evidence demonstrating a benefit for continuing visits [16]. Nonetheless, specific populations may benefit from follow-up visits [17, 18]. Our clinic is a training site for Obstetrics and Gynecology residents that recommends post-insertion follow-up visits for all women for two reasons. First, they are recommended for training purposes. Follow-up visits help us monitor both major and minor complication rates after contraceptive implant insertion are performed during residency rotations, which may be under-reported if no appointment is made. Feedback for providers can improve the competence with which they provide services. Second, almost half of our clients were adolescents and young adults, patients at increased risk of early discontinuation of contraception. Therefore, providing adequate information, performing monitoring, and helping clients deal with side effects may promote contraception continuation [18].

During the COVID-19 pandemic, healthcare services required modification to adhere to social distancing policies. During the time, telemedicine become a ‘new normal’ type of medical service provided in Thailand and our own hospital [19]. After we launched the patient follow-up program using the LINE® OA platform, we observed that our clients’ acceptance of telemedicine significantly increased over time and that this increased acceptance was associated with the peak of the COVID-19 outbreak. Approximately 50% of our clients who initiated contraceptive implantation before the peak of the outbreak chose the telemedicine modality for follow-up. During the peak of the COVID-19 outbreak, telemedicine usage increased to 65% of clients. This increase in acceptability may be explained by social adaptation during the pandemic. This may be because the general population changed their daily lifestyles and became more familiar with the use of online technology.

Telemedicine services are preferred over hospital attendance when caring COVID-19 patients. Disruptions associated with COVID-19 also changed the culture of visiting doctors for the non-urgent care of other diseases. Another reason for the increased usage of telemedicine may be the severity of the peak of the outbreak during which time the number of COVID-19 cases was greater and more severe than during the initial outbreak [13]. Similar to the usage of telemedicine for contraceptive implantation follow-up, telemedicine services accounted for 60% of postpartum visits during the peak of COVID-19 and dropped to 48% throughout the ongoing period [20]. As well as, the study on telehealth for headache care showed a 52% decline in in-person visits, while video visits significantly increased by 2.05 times and telephone visits increased by 15.2 times, compared to the period before the pandemic [21]. Studies conducted in the United States have reported telemedicine uptake rates for reproductive health services, including long-acting reversible contraception (LARC), contraceptive counseling, and STI-related issues, to be approximately 40–60% of overall visits during the COVID-19 pandemic. and were not associated with the peak of the pandemic [22, 23]. These trends in telemedicine usage across different healthcare domains imply that the severity of COVID-19 may have played a role in driving the acceptability and utilization of telemedicine. In a retrospective study examining the implementation of telemedicine as a new approach to postpartum care in Thailand during the COVID-19 pandemic, revealed that postpartum contact increased from 48.0 to 64.6% following the introduction of telemedicine [24]. As well as the study comparing telemedicine-delivered diabetes self-management education with in-person sessions in Thailand, participants in the telehealth group reported high satisfaction levels similar to those in the in-person visit group [25]. This suggests that Thais have accepted telemedicine as an alternative for follow-up care. A previous study regarding the use of telemedicine for contraceptive counseling in early phase of COVID-19 revealed that clients were highly satisfied with online services [26]. In fact, more than 70% of participants suggested that telemedicine services should be maintained after the COVID-19 pandemic. Patients generally hold a positive perception of telehealth based on various advantages it offers, such as saving time by reducing travel and waiting times, improving accessibility to healthcare services, providing greater convenience, and being cost-effective [27]. We believe that the reduced risk of COVID-19 exposure is also the reason for the increased the acceptability of telemedicine. As clients become more familiar with using telemedicine and experience its benefits, there is a potential to develop and expand the services to better meet their needs. This can contribute to the sustained utilization of telemedicine beyond the pandemic period.

Our study revealed that payment method may be associated with the use of telemedicine. Clients who received contraceptive implant services who received governmental benefits had higher telemedicine usage rates than those who self-paid. We hypothesized that clients who paid out of their pocket may have had a greater concern or need for visiting their doctors. However, data on contraceptive implant insertion were collected from regular office hours and extended hour clinics. Notably, basic characteristics of clients of the two types of clinics differ. Government benefits for contraceptive implant services are usable only at clinics that operate during normal office hours. In contrast, extended-hours clinics are privately operated and require self-payment. Patients of extended-hours clinics tended to prefer in-person versus online follow-up visits. Our hypothesis suggests that one potential reason for the increased acceptability of telemedicine services instead of in-person visits is the convenience it offers. The convenience of scheduling appointments at extended-hour clinics, without the need to take time off from work, may lead clients to prefer in-person visits. Conversely, for follow-up visits during regular office hours, the requirement to leave their jobs could influence their preference for telemedicine. No reimbursement method differences were observed between clients who participated in telehealth and those who did not, which may be explained by the fact that clients must pay around $70 for contraceptive implants if they are deemed ineligible for government benefits. Differences between reimbursement methods include service fees of approximately $3, which are omitted for individuals with universal coverage, KCMH, and government officers’ reimbursement policies. Indeed, the impact of reimbursement methods is relatively minimal when compared to the distinction between self-payment and government benefits. In our clinic, telemedicine is provided free of charge. Characteristics of clients may differ if there are cost differences associated with health insurance coverage and reimbursement for telemedicine and in-person follow-up services. This difference can function as a barrier to the adoption of telemedicine [28].

Several prior studies, conducted in the United States examining factors influencing telemedicine utilization across different types of consultations, found potential differences in race and ethnicity among those using telemedicine [23, 29, 30]. However, in this study, we did not use race or ethnicity as a coviariate because all participants were Thais. Our findings indicated a tendency towards a lower odds ratio for telehealth usage among individuals with underlying diseases. We postulate that patients are generally advised to arrange an in-person visit, which conveniently allows the post-insertion follow-up to be conducted during this same appointment. While previous studies have shown that patients in various disease domains, such as communicable and non-communicable diseases as well as orthopedics, are inclined to continue utilizing telehealth consultations after the COVID-19 pandemic,[31–36] it is noteworthy that only one-third of surgical patients expressed their willingness to continue using telemedicine [37]. This difference in acceptance may be influenced by the perception that patients have regarding the complexity of their health conditions, leading them to prefer in-person visits to meet their doctor. Nevertheless, it is important to consider that in the cultural context of the Thai population, the acceptability of telemedicine may differ from other countries, highlighting the need for further research to understand these potential variations.

Our results do not demonstrate an association between age group and telehealth usage. However, a previous study found that telemedicine usage was highest among young to middle-aged adults, particularly for urgent care [38]. This could be explained by the fact that our study population included patients of reproductive age, from adolescents to mid-adults. These populations might be familiar with using technology and adapting to telemedicine usage. The LINE® platform is the most popularly used texting application in Thailand. According to Hootsuite Analytics in 2019, approximately 84% of Thai internet users have active LINE® accounts [39]. Further, internet accessibility in Thailand increased to approximately 76% in 2020 [40].

In our study, we examined the occurrence of adverse events following contraceptive implant insertion. We found that approximately one-third of clients experienced mild ecchymosis, while a significant ecchymosis was reported by 13% of clients. Interestingly, our study showed a higher incidence of adverse events compared to previous reports from randomized controlled trials (RCTs), which reported bruising rates of around 6–7% [41]. It’s worth noting that our study assessed adverse events at 7 days post-insertion, whereas the RCTs followed up at 6 weeks. This difference in follow-up duration suggests that early follow-up may slightly increase the reporting rate of these side effects, because these side effects are transient in nature. Additionally, we hypothesize that telemedicine may facilitate easier reporting of minor concerns, and we plan to further research this aspect.

Our study had several limitations. First, the study period was limited to before the peak of the outbreak (June 2020–November 2020) and during the COVID-19 outbreak peak (December 2020–August 2021) in Thailand. Our data cannot be generalized to other timeframes that may influence the decisions of clients to use telemedicine. However, the study period may represent the telemedicine uptake rate encountered during the most serious part of the outbreak. Information regarding declining telemedicine uptake rates during pandemic resolution are lacking. Second, our service using the LINE® OA platform predominately text message-based communication. Therefore, real-time interaction with healthcare providers is infrequent. The acceptability of telemedicine may differ if other platforms are used. Third, data were extracted from medical records to assess characteristics of patients who use telemedicine. No information regarding the follow-up rate of clients who chose in-person follow-up visits were included. Therefore, no comparison of follow-up methods or reported rates of post-insertion complications were performed. Clients who participated in telemedicine follow-up visits may have had more insertion complications than those who did not participate in the service. Therefore, complication rates of this study were higher than those reported in the previous studies [41]. The retrospective design of our study also prevented us from conducting in-depth interviews to determine reasons underlying the selection of telemedicine. We were unable to demonstrate which COVID-19- or convenience-related factors influenced the increase in telemedicine usage during the peak of the pandemic. In addition, no long-term follow-up data regarding between-follow-up method differences in early discontinuation rates. Finally, our clinic setting is a tertiary referral hospital located at the center of Bangkok; therefore, data may be representative of urban communities exclusively. Most people can access the internet via their mobile phones, which are widely used among residents of the area.

A strength of our study is that baseline characteristics of all clients using both telemedicine and in-person visits were collected. Further, our telemedicine service was available free of charge, eliminating a barrier for telemedicine service access. We used a logistic regression model to analyze which confounders influenced telemedicine uptake. In addition, our study assessed use of a pioneering contraceptive service via telemedicine in Thailand; therefore, it has the potential to improve contraceptive telemedicine service development and support medical education.

Our study sheds light on the crucial role of telemedicine in contraceptive implantation services, a significant development in the healthcare landscape. Even though the focus may seem specialized, the practical implications are broad and transformative. It has provided an opportunity to introduce and familiarize clients with the use of telemedicine, highlighting its importance and potential benefits. This experience has laid the foundation for a promising future in terms of integrating and expanding telemedicine in healthcare delivery. In upcoming research, we will investigate client’s satisfaction with telemedicine and compare the continuation rates of contraceptive implants usage between those who receive post-insertion follow-up via telemedicine and those who do not. This knowledge will be instrumental in guiding the development and implementation of telemedicine services to effectively meet the diverse needs of clients. This approach ensures the sustainability of telemedicine and opens up possibilities for expanding to other contraceptive services.

## Conclusions

This study underscores telemedicine’s vital role during the COVID-19 pandemic, particularly in facilitating contraceptive implantation services. Our findings indicate a significant increase in the acceptance and utilization of telemedicine during the pandemic’s peak. The data further reveals that factors such as the timing of the second COVID-19 outbreak in Thailand, governmental benefits for contraceptive implant payment, and the timing of contraceptive implant initiation are significantly associated with telemedicine use. This insight supports the continued integration of telemedicine into healthcare practices, especially for services like family planning, where remote follow-ups can provide safe, effective, and convenient care.

## Data Availability

The datasets used and/or analysed during the current study available from the corresponding author on reasonable request.

## List of Abbreviations

LINE® OA: LINE® Official Account
LARC: Long-acting reversible contraceptives
KCMH: King Chulalongkorn Memorial Hospital
